# A Review on Glycemic Control in Type 2 Diabetes Mellitus by Arm Ergometer Exercise

**DOI:** 10.7759/cureus.27476

**Published:** 2022-07-30

**Authors:** Shubhada R Dhait, Vishnu Vardhan, Rashmi R Walke

**Affiliations:** 1 Cardiovascular and Respiratory Physiotherapy Department, Ravi Nair Physiotherapy College, Datta Meghe Institute of Medical Sciences, Wardha, IND

**Keywords:** cardiorespiratory fitness, pathophysiology, glycated haemoglobin (hba1c), physiotherapy intervention, aerobic exercise, glucose control, 6 min walk, physical activeness, arm ergometer, diabetes mellitus

## Abstract

It has been suggested that regular physical activity has become a part of rehabilitation in controlling blood glucose levels in type 2 diabetes mellitus patients. In type 2 diabetes mellitus the cells become resistant to insulin, which leads to elevated blood glucose over time and leads to prediabetes and type 2 diabetes mellitus (T2DM). The typical adult's blood contains about 5-10 grams of glucose when their blood glucose content is 100 milligrams per decilitre. About half a billion individuals are at risk for diabetes worldwide. Physical exercise has been proved to be better therapy for controlling blood glucose in persons at risk for diabetes, preventing further body complications. Three significant interests in exercising to delay the onset of T2DM. First, increased blood flow into the muscle is triggered by skeletal muscle activity, which promotes glucose absorption from the bloodstream. Second, it reduces abdominal adipose tissue, a well-known risk of metabolic disease. Third, physical exercise with moderate intensity has been proven to boost glucose uptake by 40 percent. Globally and in developing nations like India, the burden of diabetes is expanding, attributable to a rise in overweight/obesity and sedentary lifestyles. It is difficult to provide healthcare for diseases like diabetes since it requires a consistent commitment to the prescribed course of treatment. Based on the correlation between fasting plasma glucose (FPG) or hemoglobin A1c (HbA1c) and retinopathy, cut-off values for glucose and HbA1c are estimated.

## Introduction and background

Type 2 diabetes is described as an inadequate amount of insulin sensitivity over time. The disease develops when the body's glucose metabolism is disturbed. Insulin resistance is commonly triggered either by insufficiency of insulin secretion or a high protein level, both of which can cause it. Insulin resistance is a phrase used to characterize the illness known as type 2 diabetes, which is independent of insulin [1]. The incidence of type 2 diabetes mellitus (T2DM) is continuously rising due to sedentary lifestyles worldwide [2].

Three significant flaws characterize the start of hyperglycemia in T2DM: Hepatic glucose synthesis increases, insulin secretion decreases, and insulin action is hampered [3]. Diabetes is becoming more prevalent globally, especially in developing nations such as India, owing to rising rates of overweight/obesity and harmful lifestyles. According to predictions from 2019, by 2045, India's diabetes population will have grown to approximately 134 million individuals. India's population was estimated to be around 77 million. Type 2 diabetes accounts for most cases and can cause diabetes-associated problems [4]. Chronic illness management, such as diabetes, is complicated due to long-term adherence to related cure, protection, or administration consequences [5].

Based on the belief that type 2 diabetics and people with normoglycemia reduced the same amount of weight, a new study found that the amount of pancreatic fat dropped after surgical treatment. The initial insulin reaction normalized, in people with type 2 diabetes, suggesting that a fatty pancreas is linked to type 2 diabetes [6].

The pathogenesis of hyperglycemia is linked to muscle insulin resistance as opposed to hepatic insulin resistance. The differentiation was elegantly signified in type 2 diabetes trials in which modest calorie restriction decreased fat in the liver, hepatic insulin sensitivity, and fasting plasma glucose levels improved, but muscular insulin resistance remained unchanged [7].

Risk factors

Obesity emerged as the most modifiable risk factor for T2DM among all the factors evaluated. Overweight or obesity is a common occurrence in 46 % of type 2 diabetes mellitus [8]. Active and passive smoking causes a reduction in the absorption of glucose in muscles, and this effect is more common in males than in women. Depression is a disorder that directly affects a person’s acts or feelings. This elevates the sympathetic nervous system and hypothalamic-pituitary-adrenal activities which directly give rise to insulin resistance. The insulin resistance due to a decrease in the body’s glucose uptake may occur due to elevated blood pressure in the arteries in cardiovascular disease [9].

Pathophysiology

In type 2 diabetes, the body either produces insufficient insulin or the cells reject it. When cells do not properly utilise insulin in the beginning, this condition is known as insulin resistance. The pancreas' capacity to produce glucose declines as glucose consumption rises [10].

There are three critical features of exercising to delay the onset of T2DM. First, increased blood flow into the muscle is triggered by skeletal muscle activity, which promotes glucose absorption from the bloodstream [11]. Second, reduces abdominal adipose tissue, a well-known risk for metabolic diseases [12]. Third, medium physical activity has increased glucose intake by 40%. To prevent the incidence of T2DM, reducing weight has become the best treatment, which allows the cells of the body to use blood glucose more efficiently [13]. It now seems that the inflammatory process of white adipose tissue (WAT) has resulted from immune system activation that lasts a long time, leading to diabetes and other impairments in an individual [14]. The glucose transport is isotypes regulated by contractions and insulin in skeletal muscle activity via glucose transport proteins. The blood glucose levels tend to decline while performing moderate-intensity exercise in type 2 diabetes mellitus as the utilization of the blood glucose levels are raised more as compared to hepatic glucose production [15].

Features

Steadily increasing urination and thirst, lack of stamina, lethargy, bacterial and fungal infections, and delayed wound healing are the common features of T2DM [16].

Incidence

The diabetic individuals in the 2019 survey showed that 77 million were affected in India, which is predicted to get a hike of over 134 million by 2045 [4]. The geographical distribution is also related to the diabetes pattern. Due to genetic factors involving the influences of environmental factors like obesity, a sedentary lifestyle gives rise to aetiology of diabetes in India [17]. Worldwide half a billion people are affected by diabetes and the number is estimated to grow by 25% in 2030 and 51% in 2045 [18].

Investigations

The HbA1c screening or plasma glucose measure is often used to carry out the diagnosis of mellitus. The correlation of FPG or HbA1c with retinopathy is used to compute the slashed values for glucose and HbA1c [19].

Prevention

To modify the incidence of T2DM, reducing weight has become the best treatment, which allows the cells of the body to use blood glucose more efficiently [13].

## Review

The study done by Jeng et al. has shown that the effect of the arm cycle ergometer is effective in decreasing the serum glucose level in patients with diabetes. The patient who met the inclusion and exclusion criteria were involved in the study and 12 exercise sessions were scheduled where the different exercise intensities and durations of serum responses were measured and the results had been concluded that it is being beneficial in diabetic patients [20].

The article shows the significant effect of cognitive-behavioural therapy CBT) on glycemic control with consequences of insomnia in T2DM patients. Outcomes were measured before and after treatment to evaluate diabetes self-care behaviour (DSCB), glucose level, and fatigue. CBT is the most effective treatment for patients with T2DM, which alters the fundamental metabolic changes in the body and targets the physiological mechanism for controlling diabetes mellitus [21].

The randomised controlled trial (RCT) of the first year of the Sophia Step Study measured the change in physical and behavioral activity at a baseline of 6 and 12 months by accelerometry as daily steps. This was the three-armed RCT study assessing pedometers and the tools for self-considering of stepping containing and excluding by monitoring the health care professionals on cardiac condition and getting HbA1c as a primary outcome evaluated pre and post-study [22].

In a comparative study between functions of the upper extremity of subjects with T2DM and with healthy individuals, the authors included 36 diabetes subjects and 36 healthy subjects. Strength grip was assessed using a hand dynamometer, endurance was evaluated through the Unsupported Upper Limb Exercise Test (UULEX), and disability with Disabilities of Arm Shoulder and Hand Test (DASH-T) [23]. In another study, the authors randomized 25 subjects with diabetes and divided them into two groups, 13 subjects with scheduled physical exercise and 12 subjects with a usual treatment program. After one year, the study concluded that the subjects with the trained program sustained their aerobic capacity, while there was decreased capacity in other groups [24]. A study was carried out investigating the ability for functional exercise in individuals with diabetes mellitus and individuals in good health with 131 participants and concluded that a six-minute walk test (6MWT) is an effective test indicating the evaluation of daily physical activity in diabetes mellitus patients [25].

A study reviewed the various format of the studies proving the fact that upper extremity exercises help the patients to control glucose the goal was to assess the effect of Simplified Diabetes Nutrition Education (SDNE) on controlling blood sugar levels and achieving positive clinical outcomes with T2DM. Individuals who received weekly modeled diabetes nutrition for 12 weeks, including standard plan HbA1c and other metabolic parameters, diet intake, and level of physical activity were evaluated at baseline every 12 weeks and 22 weeks and showed better improvement in all the parameters involved [26].

Another study examined the influence of aerobic and strength exercise on blood glucose control in 86 people with T2DM vs usual care in a parallel-group design. The session was tracked for 12 weeks and lasted 75 min/day, two days per week. The critical outcome, HbA1c, was found to substantially influence before and after the intervention [27].

This experimental study compared the effects of Traditional and Modified Arm Swing Exercise regimens on glucose control and nutrition levels in patients with type 2 diabetes. Seventy-six type 2 diabetes individuals were separated into three groups. BMI, waist size, fat tissue, and striated muscle were examined using measuring tapes, overall health monitors, and glucose levels were monitored at baseline and after the programmes, and overnight capillary blood glucose was measured, HbA1c, and nutritional status using measuring tapes, and blood glucose monitors. Both Traditional and Modified Arm Swing Exercise groups exhibited reduced overnight capillary blood glucose, HbA1c, body mass index (BMI), waist size, and fatty tissue after the treatment, but higher skeletal muscle than before [28].

According to a study, the prevalence of T2DM and pre-diabetic disorders impaired fasting glucose (IFG) and impaired glucose tolerance (IGT) is rapidly increasing. There is considerable evidence that persons who are not physically active enough are more likely to acquire T2DM. Workout routines, when used in conjunction with other lifestyle changes, can aid persons with pre-diabetes in eliminating T2DM and optimizing glycemic control [29].

An RCT was conducted to see if the vascular nerve function is altered by a strict programme of aerobic, isokinetic strength, or a mix of aerobic and isokinetic strength exercise. Detailed nerve conduction was examined at baseline, promptly after session, and again at 12-week post-intervention as a critical end measure. The SF-36V questionnaire, as well as quantitative sensory testing and symptom-limited treadmill stress tests, were presented to all participants. Treadmill test outcomes improved in individuals who did an aerobic or combination aerobic-isokinetic strength training strategy [30].

For future research, it is important that designed studies are conducted in order to validate that blood glucose levels can be controlled through arm exercise in individuals with type 2 diabetes mellitus. 

## Conclusions

The article concluded that aerobic exercise is beneficial for controlling diabetes in an individual with T2DM. The arm ergometer exercise was found to be effective for patients with type 2 diabetes and a decrease in glucose levels. A crucial part of the pathogenesis of T2DM is played by glucose transporter 4 (GLUT4). Patients with T2DM have a barrier to glucose entry into the cell for the production of energy due to its faulty expression or translocation to the plasma membrane of peripheral cells. Patients with regular aerobic exercise had shown better control of their disease. To prevent, regulate, treat, or reverse the pathophysiology of T2DM and its problems, it is imperative to understand the mechanisms involved in each stage of the development and consequences of T2DM. Cardiorespiratory fitness had a significant improvement by the interventional strategy given to the patients with type 2 diabetes mellitus.

## References

[1] Motahari-Tabari N, Ahmad Shirvani M, Shirzad-E-Ahoodashty M, Yousefi-Abdolmaleki E, Teimourzadeh M (2014). The effect of 8 weeks aerobic exercise on insulin resistance in type 2 diabetes: a randomized clinical trial. Glob J Health Sci.

[2] Gao S, Tang J, Yi G (2021). The therapeutic effects of mild to moderate intensity aerobic exercise on glycemic control in patients with type 2 diabetes mellitus: a meta-analysis of randomized trials. Diabetes Ther.

[3] Cantley J, Ashcroft FM (2015). Q&A: insulin secretion and type 2 diabetes: why do β-cells fail?. BMC Biol.

[4] Pradeepa R, Mohan V (2021). Epidemiology of type 2 diabetes in India. Indian J Ophthalmol.

[5] Mathur P, Leburu S, Kulothungan V (2022). Prevalence, awareness, treatment and control of diabetes in India from the countrywide National NCD Monitoring Survey. Front Public Health.

[6] Heiskanen MA, Motiani KK, Mari A (2018). Exercise training decreases pancreatic fat content and improves beta cell function regardless of baseline glucose tolerance: a randomised controlled trial. Diabetologia.

[7] Taylor R (2012). Insulin resistance and type 2 diabetes. Diabetes.

[8] Vijayakumar G, Manghat S, Vijayakumar R (2019). Incidence of type 2 diabetes mellitus and prediabetes in Kerala, India: results from a 10-year prospective cohort. BMC Public Health.

[9] Ismail L, Materwala H, Al Kaabi J (2021). Association of risk factors with type 2 diabetes: a systematic review. Comput Struct Biotechnol J.

[10] (2022). Pathophysiology of Diabetes Mellitus. https://www.kindredhealthcare.com/resources/blog-kindred-continuum/2013/11/07/pathophysiology-of-diabetes-mellitus..

[11] Venkatasamy VV, Pericherla S, Manthuruthil S, Mishra S, Hanno R (2013). Effect of physical activity on insulin resistance, inflammation and oxidative stress in diabetes mellitus. J Clin Diagn Res.

[12] Strasser B (2013). Physical activity in obesity and metabolic syndrome. Ann N Y Acad Sci.

[13] (2019). 3. Prevention or Delay of Type 2 Diabetes: Standards of Medical Care in Diabetes-2019. Diabetes Care.

[14] Bastard JP, Maachi M, Lagathu C (2006). Recent advances in the relationship between obesity, inflammation, and insulin resistance. Eur Cytokine Netw.

[15] Colberg SR, Sigal RJ, Fernhall B (2010). Exercise and type 2 diabetes: the American College of Sports Medicine and the American Diabetes Association: joint position statement. Diabetes Care.

[16] Goyal R, Jialal I (2022). Diabetes Mellitus Type 2.

[17] Kaveeshwar SA, Cornwall J (2014). The current state of diabetes mellitus in India. Australas Med J.

[18] Saeedi P, Petersohn I, Salpea P (2019). Global and regional diabetes prevalence estimates for 2019 and projections for 2030 and 2045: results from the International Diabetes Federation Diabetes Atlas, 9th edition. Diabetes Res Clin Pract.

[19] Kharroubi AT, Darwish HM (2015). Diabetes mellitus: the epidemic of the century. World J Diabetes.

[20] Jeng C, Chang WY, Chen SR, Tseng IJ (2002). Effects of arm exercise on serum glucose response in type 2 DM patients. J Nurs Res.

[21] Alshehri MM, Alothman SA, Alenazi AM (2020). The effects of cognitive behavioral therapy for insomnia in people with type 2 diabetes mellitus, pilot RCT part II: diabetes health outcomes. BMC Endocr Disord.

[22] Rossen J, Hagströmer M, Yngve A, Brismar K, Ainsworth B, Johansson UB (2021). Process evaluation of the Sophia Step Study- a primary care based three-armed randomized controlled trial using self-monitoring of steps with and without counseling in prediabetes and type 2 diabetes. BMC Public Health.

[23] Yerli̇kaya T, Çalik BB, Cavlak U, Si̇rkeci̇ Ö (2021). Upper Extremity Functioning in Individuals with Type 2 Diabetes Mellitus: A Comparative Study. Clin Exp Health Sci.

[24] Brun J-F, Myzia J, Bui G, Grubka E, Karafiat M, Mercier J, Raynaud de Mauverger E (2020). The 6-minute walk-test in type 2 diabetics predicts to some extent maximal aerobic capacity but not its training-induced improvement. Ann Musculoskelet Med.

[25] Kuziemski K, Słomiński W, Jassem E (2019). Impact of diabetes mellitus on functional exercise capacity and pulmonary functions in patients with diabetes and healthy persons. BMC Endocr Disord.

[26] Hashim SA, Mohd Yusof BN, Abu Saad H, Ismail S, Hamdy O, Mansour AA (2021). Effectiveness of simplified diabetes nutrition education on glycemic control and other diabetes-related outcomes in patients with type 2 diabetes mellitus. Clin Nutr ESPEN.

[27] Ranasinghe C, Devage S, Constantine GR, Katulanda P, Hills AP, King NA (2021). Glycemic and cardiometabolic effects of exercise in South Asian Sri Lankans with type 2 diabetes mellitus: A randomized controlled trial Sri Lanka diabetes aerobic and resistance training study (SL-DARTS). Diabetes Metab Syndr.

[28] Terathongkum S, Phonyiam R, Koonmee P (2021). Effects of traditional and modified arm swing exercise programs on blood glucose and nutritional status among people with type 2 diabetes: a secondary data analysis. Pac Rim Int J Nurs Res.

[29] Hordern MD, Dunstan DW, Prins JB, Baker MK, Singh MA, Coombes JS (2012). Exercise prescription for patients with type 2 diabetes and pre-diabetes: a position statement from Exercise and Sport Science Australia. J Sci Med Sport.

[30] Stubbs EB, Fisher MA, Miller CM, Jelinek C, Butler J, McBurney C, Collins EG (2019). Randomized controlled trial of physical exercise in diabetic veterans with length-dependent distal symmetric polyneuropathy. Front Neurosci.

